# Supplementary material for: Chromothripsis during telomere crisis is independent of NHEJ, and consistent with a replicative origin
Source: Genome Res. 2019 May;29(5):737–49. doi: 10.1101/gr.240705.118 (PMC6499312; doi:10.1101/gr.240705.118)
Supplement: Supplemental Material [file supp_gr.240705.118_Supplemental_file_1.zip › contigs/annotated_contigs/DB76/contig.2.DB76_length_282_mean_cov_15.1489361702.docx]

**DB76_length_282_mean_cov_15.1489361702**

```
TCATCACTGCAGAAGATGAAATAACTGTTTAACCTGGTCATGATGAACCATTTAGCTGAGATTCCCCAACCTTACACCAAGCCATTCTA
 >chr12:65211782-65211950 - E=4e-79
AGATTTTTTTTTTTTGGTAGAGTCTCACTCTGTTGCCCAGGCTGGAGTACAGTGGAGGGATGTCGGCTCA|CTG|GGTTTCAGTTTCCT
                                                                       >chr12:65211032-
CATCTTTAGAATAATGATATTTGAGTTACGGTGAGAATTAAATGACATAATGAAAGTCATGGCATAAAGCAGGCGCTCAATACTGTCCT
65211155 - E=9e-63
TTGATTAGTGCTTGTTG
```
